# Case Report: A Novel Point Mutation of *SOX3* in a Subject With Growth Hormone Deficiency, Hypogonadotrophic Hypogonadism, and Borderline Intellectual Disability

**DOI:** 10.3389/fendo.2022.810375

**Published:** 2022-02-28

**Authors:** Jing Li, Yuxia Zhong, Tao Guo, Yerong Yu, Jianwei Li

**Affiliations:** ^1^ Department of Endocrinology and Metabolism, West China Hospital of Sichuan University, Chengdu, China; ^2^ Department of Respirology, West China Hospital of Sichuan University, Chengdu, China; ^3^ Department of Endocrinology, Hongya County People’s Hospital, Meishan, China

**Keywords:** *SOX3*, frame-shift mutation, growth hormone deficiency, hypogonadotrophic hypogonadism, intellectual disability

## Abstract

*SOX3* is critical for the development of the pituitary, brain, and face, and *SOX3* mutations may lead to hypopituitarism, intellectual disability, and craniofacial abnormalities. Common *SOX3* mutations are duplications and deletions of the whole or part of *SOX3*, yet only a few cases with point mutations were reported by far. We present a case with growth retardation, small penis, and learning difficulty. Further assessment confirmed growth hormone deficiency, hypogonadotropic hypogonadism (HH), and borderline intellectual disability. He also responded well to gonadotropin-releasing hormone stimulation test, which suggests defects in the hypothalamus, contrary to previous studies that reported defects in the pituitary. A pathogenic frame-shift mutation of *SOX3* was found. A heterogeneous missense mutation in *SEMA3A* was identified in this patient as well, which may also contribute to the development of HH. As far as we know, this is the first report that a frame-shift mutation of *SOX3* constitutes rare genetic causes of HH and growth hormone deficiency. Whether mutations in these two genes act synergistically in the pathogenesis of the patient’s phenotype remains to be further investigated. We believe that our case extends the phenotypic spectrum and genetic variability of *SOX3* mutation.

## Introduction

*SOX3* is a member of the SOX (SRY-related high-mobility group box) family of transcription factors located on the X chromosome and is expressed in the developing central nervous system from early stages of development (1, 2). It has a short N-terminal domain and a longer C-terminal domain containing four polyalanine tracts involved in transcriptional activation (1). *SOX3* is critical for normal development of the pituitary, brain, and face in both mice and humans (3). A range of phenotypes are associated with *SOX3* mutations in humans, including hypopituitarism ranging from isolated growth hormone deficiency to panhypopituitarism, intellectual disability, and craniofacial abnormalities (4). Almost all the subjects developed clinical symptoms before puberty. Common *SOX3* mutations are duplications and deletions of the whole or part of *SOX3*, yet point mutations of *SOX3* are rarely reported. This is the first report of a *SOX3* frame-shift mutation in a patient with growth retardation, delayed puberty, and learning difficulty.

## Case Description

**Figure 1:**
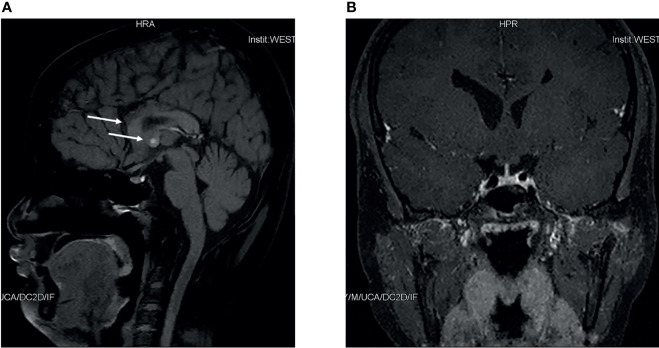
Pituitary magnetic resonance imaging of this patient. Sagittal magnetic resonance imaging scans of this patient **(A)** indicated dysgenesis of the corpus callosum, and a small nodule in septum pellucidum. Coronal scans of this patient **(B)** indicated nothing abnormal.

**Figure 2:**
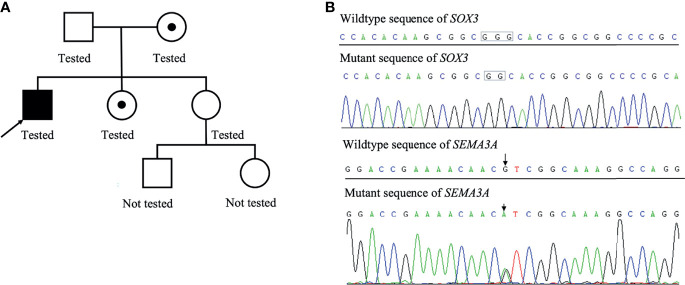
Family pedigree of this patient **(A)**. The patient’s mother was the carrier of a heterogeneous mutation in SOX3 and SEMA3A, and one of his elder sister was the carrier of a heterogeneous mutation in SEMA3A. Results of whole-exome sequencing of the proband **(B)**.

A Chinese boy was found to have short stature and poor performance at school when he was 7 years old. He was born at full term after an uncomplicated pregnancy. His parents were non-consanguineous. He was diagnosed with omphalitis 2 days after birth and recovered after being treated with antibiotics. The patient’s mother was not sure about his mental and physical development before 7 years of age. So he went to a local hospital, yet diagnosis and treatment were unknown, and no improvement was achieved. At the age of 14 years, he was found to have gynecomastia and was admitted to the West China Second University Hospital. He was 147 cm tall and 38.5 kg at that time. Further evaluations revealed that he had normal estradiol (27.6 pg/ml, reference range: 0–39.8), low testosterone (0.23 ng/ml, reference range: 2.41–8.27), low luteinizing hormone (LH; 1.4 IU/l, reference range: 1.5–9.3), and normal follicular stimulating hormone (FSH; 2.5 IU/L, reference range: 1.4–18.1). His growth hormone (GH) was 0.50 ng/ml, and the insulin-like growth factor was 297.0 ng/ml (reference range: 221–904 ng/ml for subjects aged 13–18 years). The GH stimulation test confirmed GH deficiency because the peak GH after stimulation was 2.40 ng/ml, yet we were not sure how the GH stimulation test was performed. He had normal thyroid and adrenocortical function. He was administered recombinant GH 4IU per day consecutively for 3 months, and later for the inconvenience of injection, he used recombinant GH irregularly. We were not sure about the dose and frequency of recombinant GH he used after the first 3 months. His height increased from 147 to 160 cm in 2 years, and there was no change in the recent 2 years. He was admitted to the West China Hospital complaining of gynecomastia and small penis at the age of 18 years. Physical examination showed that he was 160 cm tall and 54 kg, had a high-pitched voice, and hand span of 162 cm. Pubic hair and external genitalia development were both stage I in the Tanner stage. Bilateral breast enlargement was found without galactorrhea or overlying skin changes. His olfaction was normal. Complete blood count, biochemical profiles, and urinalysis showed nothing remarkable. Random measurement of sex hormone indicated very low testosterone and decreased LH and FSH. Prolactin level and thyroid function were normal. Adrenocorticotropic hormone (ACTH) was 21.10 ng/l, and morning cortisol was 291 nmol/l. Bone age was 14 years, which was considerably delayed. The gonadotropin-releasing hormone (GnRH) stimulation test using 100 μg gonadorelin intravenously showed that the peak LH was 11.3 mIU/ml at 60 min, compared with 1.0 mIU/ml at baseline. The repetitive GnRH loading test was performed in which 100 μg gonadorelin was administered intravenously for 7 consecutive days, and in day 7, another standard GnRH stimulation test was performed (**Table 1**). The peak LH was 44.6 mIU/ml at 30 min. To further evaluate the pituitary adrenal axis and GH secretion, the GH stimulation test by insulin-induced hypoglycemia showed poor GH reserve (peak 1.87 ng/ml) and slightly blunted adrenocortical reserve, with the peak cortisol to be 487 nmol/l (**Table 2**). Human chorionic gonadotropin (HCG) was administered 2,000 IU per day intravenously for 3 consecutive days, and fasting blood was drawn on the 4th day which indicated that testosterone increased from 0.06 to 2.06 ng/ml. Magnetic resonance imaging (MRI) demonstrated nothing abnormal in the sellar area, dysgenesis of the corpus callosum, and a small nodule in the septum pellucidum (**Figure 1**). Cognitive assessment using full-scale intelligence quotient showed a score of 72, which was borderline, very close to intellectual disability. Whole-exome sequencing indicated a hemizygous frame-shift mutation of *SOX3* (c.287 delG, p.G96Afs*44) located in chromosome X, resulting in the change of 44 amino acid residues between codons 96 and 139 (**Figure 2**). The mutation was highly pathogenic, and this variant was not found in the Exome Aggregation Consortium, dbSNP, 1000 Genomes project, or in-house databases. A heterogeneous point mutation of *SEMA3A* (c.2198G>R) was also identified by Sanger sequencing, which resulted in a missense mutation (p.R733H). His mother was confirmed to have the same mutation in *SOX3* and *SEMA3A*, and one of his elder sisters had the same mutation in *SEMA3A.* Sanger sequencing of his father and the other elder sister turned negative. His mother had a history of schizophrenia and was well-controlled by antipsychotic medication. Otherwise, his mother was quite healthy. She reached her target height, had normal menstruation before menopause, and gave birth to two daughters and one son after natural conception. Although a cognitive assessment was not performed, she behaved normally and had no difficulty in working and taking care of her children. His father and two elder sisters were phenotypically normal. For the treatment of this patient, the short-term goal was to help him achieve puberty induction and sexual maturation, and the long-term goal was to achieve induction of fertility. Since testosterone replacement does not restore normal spermatogenesis in men with GnRH deficiency, and fertility was desired by the patient, he was prescribed 1,500 IU of HCG twice a week after discharge. He was not administered with GnRH since GnRH was very expensive. For the short follow-up time after administration of HCG, we were not sure whether he responded well to the treatment.

**Table 1:** Level of luteinizing hormone (mIU/mL) after the gonadotropin-releasing hormone loading test.

Time (min)	-15	0	15	30	60	120
Day 1	1.0	1.4	8.3	10.9	11.3	9.1
Day 7	1.9	1.9	36.9	44.6	37.1	21.1

Gonadotropin-releasing hormone (100 μg/day) was given for 7 consecutive days, and standard gonadotropin-releasing hormone stimulation test was performed in days 1 and 7.

**Table 2:** Insulin-induced hypoglycemia test.

Time (min)	0	25	30	45	60	90	120
Glucose (mmol/L)	4.70	2.51	2.70	3.48	4.05	4.65	4.84
ACTH (ng/L)	16.01	60.06	109.40	248.90	111.60	51.42	30.84
Cortisol (nmol/L)	173	224	276	425	474	487	378
Growth hormone (ng/mL)	0.97	1.86	1.87	1.08	0.78	1.08	0.93

ACTH, adrenocorticotropic hormone.

Insulin (0.15 IU/kg) was injected, and glucose, ACTH, cortisol, and growth hormone were measured at times indicated.

## Discussion

This patient presented with GH deficiency, hypogonadotropic hypogonadism (HH) due to GnRH deficiency, and borderline intellectual disability possibly caused by a novel frame-shift mutation of *SOX3*. HH is a common symptom in patients with *SOX3* mutation for the defect of the anterior pituitary gland, but very few subjects developed this symptom due to GnRH deficiency. A heterogeneous missense mutation in *SEMA3A* was identified in this patient as well, which may also contribute to the development of HH. Besides, as far as we know, this case indicates for the first time that a frame-shift mutation of *SOX3* constitutes rare genetic causes of HH and GH deficiency. We believe that our case extends the phenotypic spectrum and genetic variability of *SOX3* mutation. As far as we know, no subjects have been reported to carry mutations in *SOX3* and *SEMA3A* at the same time before. Whether mutations in these two genes act synergistically in the pathogenesis of his phenotype remains to be further investigated. It also reminds pediatricians that *SOX3* mutation constitutes a rare cause of growth restriction and puberty delay.

The clinical phenotype varies dramatically in patients with *SOX3* mutations. Studies even indicated interfamilial phenotypic variability with identical mutation (5). GH deficiency was reported to be the commonest pituitary hormone deficiency seen in patients with *SOX3* mutation, and very few patients may be unaffected (6). Intellectual disability has been reported in most cases with *SOX3* mutation, although the extent of the disability varies a lot (6). Besides being involved in the hypothalamus–pituitary axis formation, *SOX3* is expressed throughout the central nervous system development and is involved in processes like pluripotency maintenance in self-renewing stem cells, lineage progression, and terminal differentiation (7). This may explain the coexistence of hypopituitarism and intellectual disability in patients with *SOX3* mutations. Some subjects may have early onset of hypoglycemia and hypothyroidism. If not treated timely and properly, this may also contribute to intellectual disability.

Semaphorin (SEMA) has an important role in nerve development. Mutations in *SEMA* and its receptors were found to affect the migration of GnRH neurons, which may lead to idiopathic HH (IHH) (8). Mutation of *SEMA3A* in this case was previously identified in a subject with Kallmann syndrome, and cells harboring this gene mutation had impaired signaling activity of the secreted protein semaphoring-3A (9). This suggests that the point mutation of *SEMA3A* has a pathogenic effect, which may explain the coexistence of IHH in this subject. However, studies also suggest that monoallelic mutations in *SEMA3A* may not be sufficient to cause the disease phenotype, since these missense mutations were also reported in the Exome Variant Server database and found in healthy control individuals (9, 10). The mutation of *SEMA3A* was also found in the NHLBI GO Exome Sequencing Project database, with minor allele frequency of 0.01%. Consistent with this finding, his mother and elder sister who harbored the same mutation are phenotypically normal. Therefore, we think that the *SOX3* mutation in this patient is highly likely to be causative for his phenotypes. Various studies have found that many subjects carrying *SEMA3A* variants also harbor other gene variants, such as *KAL1*, *PROKR2*, *PROK2*, and *FGFR1 (* 11). This suggests that *SEMA3A* may act synergistically with other genes in the pathogenesis of IHH. By far, no subjects have been reported to carry mutations in *SOX3* and *SEMA3A* at the same time before. Whether mutations in these two genes act synergistically in the pathogenesis of the phenotype in this case remains to be further investigated.

Gonadotropin deficiency was presumed to be common as well supported by small genitalia in many patients, yet the hypothalamus–pituitary–testis axis was not fully evaluated since most patients reported had not reached the age of pubertal development. Patients with *SOX3* mutation may only present with delayed pubertal development as one patient with hemizygous in-frame deletion of polyalanine tract in *SOX3* was found to have isolated GnRH deficiency (12). Although this patient had short stature and hypoplastic anterior pituitary by MRI, endocrine evaluation showed grossly normal pituitary function except for HH (13). For patients with combined pituitary deficiency including HH, we may presume that HH was due to defects in the anterior pituitary gland, namely, patients should have no response to the GnRH stimulating test. However, the patient with isolated HH and *SOX3* mutation responded well by GnRH stimulating test (13), suggesting the defect of hypothalamus. Consistently, our case also suggests that the delayed pubertal development was due to GnRH deficiency, even in the presence of growth hormone deficiency. This may be explained by an animal study that defects possibly existed in the hypothalamus as well as pituitary in *SOX3* null mice; therefore, hypopituitarism in patients with *SOX3* mutation may be due to defects in both pituitary and hypothalamus (4). Clinicians may easily assume that delayed pubertal development is due to defects in the pituitary in patients with combined pituitary hormone deficiency and *SOX3* mutation. This case reminds us of the importance to perform the GnRH stimulating test in this clinical scenario, and treatment strategies can be different in patients with defects in pituitary and hypothalamus.

For patients with clinical symptoms due to *SOX3* mutation, the most common genetic alterations reported by far were duplications and deletions of *SOX3 (* 3, 14). Therefore, the correct gene dosage of *SOX3* was believed to be critical for the normal development of hypothalamo-pituitary axis and intellectual ability (15). Polyalanine tract expansion and deletions of *SOX3*, which belongs to intragenic mutations, were also common. Yet point mutation, another kind of intragenic mutation of *SOX3*, was rarely reported. As far as we know, only three point mutations and one polymorphism, including 7 subjects, were reported by far (15–18). All of them belong to missense mutation, and this is the first report of a *SOX3* frame-shift mutation. Features of the 7 subjects as well as the subject reported in this case are summarized in **Table 3**. All the subjects were male. Like patients with other kinds of *SOX3* mutation, the most common phenotype was GH deficiency. Six patients had combined pituitary deficiency, and most subjects had confirmed intellectual disability or learning difficulties. At least 6 of them had other dysmorphic disorders, such as ophthalmological abnormalities and dental anomalies. All 8 subjects had abnormal MRI findings, with anterior pituitary hypoplasia or absence being the most common abnormality.

**Table 3:** Summary of features in all the patients with point mutation of SOX-3.

Patient number	Patient 1	Patient 2	Patient 3	Patient 4	Patient 5	Patient 6	Patient 7	Patient in this case
Gender	Male	NA	Male	Male	Male	Male	Male	Male
Age at presentation (years)	1.8	0	11.2	11.2	6	NA	NA	7
GH deficiency	Yes	NA	Yes	Yes	Yes	Yes	Yes	Yes
TSH deficiency	Yes	Yes	No	No	Yes	Yes	No	No
ACTH deficiency	No	Yes	No	No	No	No	No	No
LH/FSH deficiency	Yes	NA	No	No	Yes	Yes	Yes	Yes
Intellectual disability	No	NA	NA, but had mild learning difficulties	NA, but had mild learning difficulties	Mild	Mild	Mild	Borderline
Other features	A single central maxillary incisor	NA	Astigmatism and myopia	Bilateral cryptorchidism and astigmatism and myopia	Ophthalmological abnormalities, facial dysmorphology, dental anomalies in the maxillary midline, microcephaly, microphthalmia, short fingers and toes	Ophthalmological abnormalities, facial dysmorphology and dental anomalies in the maxillary midline	Ophthalmological abnormalities, facial dysmorphology and dental anomalies with a solitary median maxillary incisor	No
MRI imaging	Anterior pituitary hypoplasia, normal posterior pituitary, stalk intact	Hypoplastic anterior pituitary and corpus callosum, undescended posterior pituitary and absent infundibulum	Small anterior pituitary, hypoplastic pituitary stalk, ectopic posterior pituitary	Anterior pituitary and hypoplastic pituitary stalk, ectopic posterior pituitary, corpus callosum hypogenesis, midline lipoma	Persisting craniopharyngeal canal, no pituitary gland or fusion of the optic chiasm, hypoplastic intracranial optic nerves, absent optic nerves in orbit	Normal chiasm, dysplastic pituitary gland, hypoplastic optic nerves	Ectopic pituitary gland, no fusion of the chiasm, hypoplastic optic nerves, no nerve in the left orbit	Normal pituitary gland, dysgenesis of the corpus callosum, and a small nodule in septum pellucidum
SOX-3 mutation	Missense mutation, c.14G>A, p.R5Q	Missense mutation, c.127G→A, p.A43T	Missense mutation, c.424C>A, p.P142T	Missense mutation, c.424C>A, p.P142T	Missense mutation, c.449C>A, p.S150Y	Missense mutation, c.449C>A, p.S150Y	Missense mutation, c.449C>A, p.S150Y	c.287 delG, p.G96Afs*44

NA, not applicable.

In conclusion, we report a very rare *SOX3* frame-shift mutation in a Chinese male patient with a growth hormone deficiency, HH due to GnRH deficiency, and borderline intellectual disability. Although no further study was conducted to examine this mutation on the function of *SOX3*, we conclude that this variant is likely the cause of the symptoms in this patient based on the current literature and the assumption that frame-shift mutation is highly pathogenic. A heterogeneous missense mutation in *SEMA3A* may also contribute to the development of HH. Whether mutations in these two genes act synergistically in the pathogenesis of the phenotype in this case remains to be further investigated. This case extends the phenotypic spectrum and genetic variability of *SOX3* mutation. Moreover, it is essential to perform the GnRH stimulating test in patients with *SOX3* mutation and HH.

## Data Availability Statement

The original contributions presented in the study are included in the article/supplementary material, further inquiries can be directed to the corresponding author/s.

## Ethics Statement

Written informed consent was obtained from the minor(s)’ legal guardian/next of kin for the publication of any potentially identifiable images or data included in this article.

## Author Contributions

All the authors have contributed significantly. JL, YZ, and TG collected clinical data. YY helped with the interpretation of clinical data. JL wrote the manuscript. JwL revised the manuscript. All authors contributed to the article and approved the submitted version.

## Funding

The study was supported by the Science and Technology Department of Sichuan Province (Grant No. 2018HH0065 to JwL, and 2019YFS0302 to JL) and the 1.3.5 Project for Disciplines of Excellence-clinical Research Incubation Project, West China Hospital, Sichuan University (Grant No. 2020HXFH034 to JwL).

## Conflict of Interest

The authors declare that the research was conducted in the absence of any commercial or financial relationships that could be construed as a potential conflict of interest.

## Publisher’s Note

All claims expressed in this article are solely those of the authors and do not necessarily represent those of their affiliated organizations, or those of the publisher, the editors and the reviewers. Any product that may be evaluated in this article, or claim that may be made by its manufacturer, is not guaranteed or endorsed by the publisher.

## References

[1] Stevanovlć M Lovell-Badge R Collignon JM Goodfellow PN. SOX3 is an X-Linked Gene Related to SRY. Hum Mol Genet (1993) 2(12):2013–18. doi: 10.1093/hmg/2.12.2013 8111369

[2] Collignon J Sockanathan S Hacker A Cohen-Tannoudji M Norris D Rastan S. A Comparison of the Properties of Sox-3 With Sry and Two Related Genes, Sox-1 and Sox-2. Development (1996) 122(2):509–20. doi: 10.1242/dev.122.2.509 8625802

[3] Elizabeth MSM Verkerk A Hokken-Koelega ACS Verlouw JAM Argente J Pfaeffle R. Congenital Hypopituitarism in Two Brothers With a Duplication of the 'Acrogigantism Gene' GPR101: Clinical Findings and Review of the Literature. Pituitary (2021) 24(2):229–41. doi: 10.1007/s11102-020-01101-8 PMC7966638 33184694

[4] Rizzoti K Brunelli S Carmignac D Thomas PQ Robinson IC Lovell-Badge R. SOX3 is Required During the Formation of the Hypothalamo-Pituitary Axis. Nat Genet (2004) 36(3):247–55. doi: 10.1038/ng1309 14981518

[5] Burkitt Wright EMM Perveen R Clayton PE Hall CM Costa T Procter AM. X-Linked Isolated Growth Hormone Deficiency: Expanding the Phenotypic Spectrum of SOX3 Polyalanine Tract Expansions. Clin Dysmorphol (2009) 18(4):218–21. doi: 10.1097/MCD.0b013e32832d06f0 PMC2763399 19654509

[6] Arya VB Chawla G Nambisan AKR Muhi-Iddin N Vamvakiti E Ajzensztejn M. Xq27.1 Duplication Encompassing SOX3: Variable Phenotype and Smallest Duplication Associated With Hypopituitarism to Date - A Large Case Series of Unrelated Patients and a Literature Review. Horm Res Paediatr (2019) 92(6):382–89. doi: 10.1159/000503784 31678974

[7] Salemi M Romano C Ragusa L Di Vita G Salluzzo R Oteri I. A New 6-Bp SOX-3 Polyalanine Tract Deletion Does Not Segregate With Mental Retardation. Genet Test (2007) 11(2):124–27. doi: 10.1089/gte.2006.0510 17627381

[8] Cariboni A Hickok J Rakic S Andrews W Maggi R Tischkau S. Neuropilins and Their Ligands are Important in the Migration of Gonadotropin-Releasing Hormone Neurons. J Neurosci (2007) 27(9):2387–95. doi: 10.1523/JNEUROSCI.5075-06.2007 PMC6673474 17329436

[9] Hanchate NK Giacobini P Lhuillier P Parkash J Espy C Fouveaut C. SEMA3A, a Gene Involved in Axonal Pathfinding, Is Mutated in Patients With Kallmann Syndrome. PLoS Genet (2012) 8(8):e1002896. doi: 10.1371/journal.pgen.1002896 22927827 PMC3426548

[10] Kansakoski J Fagerholm R Laitinen EM Vaaralahti K Hackman P Pitteloud N. Mutation Screening of SEMA3A and SEMA7A in Patients With Congenital Hypogonadotropic Hypogonadism. Pediatr Res (2014) 75(5):641–4. doi: 10.1038/pr.2014.23 24522099

[11] Dai W Li JD Wang X Zeng W Jiang F Zheng R. Discovery of a Novel Variant of SEMA3A in a Chinese Patient With Isolated Hypogonadotropic Hypogonadism. Int J Endocrinol (2021) 2021:7752526. doi: 10.1155/2021/7752526 34721574 PMC8553509

[12] Kim JH Seo GH Kim G-H Huh J Hwang IT Jang JH. Targeted Gene Panel Sequencing for Molecular Diagnosis of Kallmann Syndrome and Normosmic Idiopathic Hypogonadotropic Hypogonadism. Exp Clin Endocrinol Diabetes (2018) 127(08):538–44. doi: 10.1055/a-0681-6608 30216942

[13] Izumi Y Suzuki E Kanzaki S Yatsuga S Kinjo S Igarashi M. Genome-Wide Copy Number Analysis and Systematic Mutation Screening in 58 Patients With Hypogonadotropic Hypogonadism. Fertil Steril (2014) 102(4):1130–6.e3. doi: 10.1016/j.fertnstert.2014.06.017 25064402

[14] Alatzoglou KS Azriyanti A Rogers N Ryan F Curry N Noakes C. SOX3 Deletion in Mouse and Human Is Associated With Persistence of the Craniopharyngeal Canal. J Clin Endocrinol Metab (2014) 99(12):E2702–E08. doi: 10.1210/jc.2014-1160 25140394

[15] Alatzoglou KS Kelberman D Cowell CT Palmer R Arnhold IJ Melo ME. Increased Transactivation Associated With SOX3 Polyalanine Tract Deletion in a Patient With Hypopituitarism. J Clin Endocrinol Metab (2011) 96(4):E685–90. doi: 10.1210/jc.2010-1239 21289259

[16] Jelsig AM Diness BR Kreiborg S Main KM Larsen VA Hove H. A Complex Phenotype in a Family With a Pathogenic SOX3 Missense Variant. Eur J Med Genet (2018) 61(3):168–72. doi: 10.1016/j.ejmg.2017.11.012 29175558

[17] Woods KS Cundall M Turton J Rizotti K Mehta A Palmer R. Over- and Underdosage of SOX3 Is Associated With Infundibular Hypoplasia and Hypopituitarism. Am J Hum Genet (2005) 76(5):833–49. doi: 10.1086/430134 PMC1199372 15800844

[18] Yu T Chang G Cheng Q Yao R Li J Yu Y. Increased Transactivation and Impaired Repression of Beta-Catenin-Mediated Transcription Associated With a Novel SOX3 Missense Mutation in an X-Linked Hypopituitarism Pedigree With Modest Growth Failure. Mol Cell Endocrinol (2018) 478:133–40. doi: 10.1016/j.mce.2018.08.006 30125608

